# Successful management of iliac artery perforation during peripheral angioplasty: a case report

**DOI:** 10.1093/ehjcr/ytaf126

**Published:** 2025-03-20

**Authors:** Saroj Kumar Sahoo, Debasis Panda, Debasis Acharya, Ramachandra Barik, Subhas Pramanik

**Affiliations:** Department of Cardiology, All India Institute of Medical Science, Sijua, Patrapada, Bhubaneswar, Odisha 751019, India; Department of Cardiology, All India Institute of Medical Science, Sijua, Patrapada, Bhubaneswar, Odisha 751019, India; Department of Cardiology, All India Institute of Medical Science, Sijua, Patrapada, Bhubaneswar, Odisha 751019, India; Department of Cardiology, All India Institute of Medical Science, Sijua, Patrapada, Bhubaneswar, Odisha 751019, India; Department of Cardiology, All India Institute of Medical Science, Sijua, Patrapada, Bhubaneswar, Odisha 751019, India

**Keywords:** Peripheral artery disease, Iliac artery perforation, Angioplasty complications, Case report, Covered stent, Balloon tamponade, Vascular intervention

## Abstract

**Background:**

Peripheral artery disease is commonly managed with percutaneous balloon angioplasty, now the standard for replacing open surgical methods. Although generally effective, this procedure can lead to complications, including the rare but potentially fatal vessel perforation. Prompt identification and intervention are crucial to avoid severe outcomes. This case report presents a rare instance of iliac artery perforation during angioplasty, successfully managed with a covered stent.

**Case summary:**

A 58-year-old male with poorly controlled type 2 diabetes mellitus presented with a non-healing ulcer and claudication in the left lower limb. Computed tomography (CT) angiography revealed complete occlusion of the left external iliac artery and significant stenosis of the right external iliac artery. During peripheral angioplasty, vessel perforation occurred after post-stenting balloon dilation, causing haemodynamic instability. Immediate management included balloon tamponade and deployment of a covered stent to seal the perforation. The patient's condition stabilized, and he was discharged with medications, including antiplatelets, anticoagulants, and statins. A follow-up CT angiography one month later showed a patent stent with good distal blood flow, and the patient remained clinically well.

**Discussion:**

Iliac artery perforation, though rare, is a severe complication of peripheral angioplasty demanding prompt and effective intervention to avert mortality and morbidity. This case demonstrates successful management using a covered stent, emphasizing the necessity of meticulous pre-procedural planning and intraoperative vigilance. The report also highlights the value of intravascular imaging and advanced techniques for vascular bed preparation in minimizing the risk of such complications during angioplasty.

**Learning points**

- Iliac artery perforation during peripheral angioplasty, although uncommon, can cause life-threatening complications, necessitating prompt detection and treatment.
- Deploying a covered stent effectively seals arterial perforations, stabilizes the patient, and prevents additional complications.
- Pre-procedural planning, with correct balloon sizing and intravascular imaging, is crucial for minimizing vessel perforation risk during angioplasty.

## Introduction

Percutaneous balloon angioplasty is widely recognized as the standard of care for managing peripheral artery disease (PAD), offering a minimally invasive alternative to open surgical procedures.^1^ This technique has revolutionized PAD treatment by enabling precise intervention with reduced recovery times and lower complication rates.^2^ Despite its widespread adoption and success, percutaneous angioplasty is associated with risks, particularly in complex cases involving high-risk patients. One of the rare but serious complications associated with this procedure is vessel perforation, which has an incidence of 0.8% to 0.9% and can result in life-threatening haemorrhage and haemodynamic instability.^3,4^

Vessel perforation during angioplasty is a critical event that demands immediate and effective intervention. Operator-related factors such as oversized balloon use, aggressive guidewire manipulation, and balloon overexpansion, alongside patient-related factors including poorly controlled type 2 diabetes mellitus (T2DM), chronic kidney disease (CKD), arterial calcification, and tortuosity, can contribute to this risk.^5^ Typical management of vessel perforation includes strategies such as balloon tamponade, stent-graft placement, or embolization, depending on the severity and location of the injury. Early recognition and timely intervention are essential to mitigate complications.

This report presents a rare case of iliac artery perforation during peripheral angioplasty in a patient with poorly controlled T2DM and CKD that was successfully managed using a commercially available covered stent. This case highlights the importance of early recognition and timely intervention in such emergencies, reinforcing the value of standardized protocols and advanced endovascular techniques in minimizing patient morbidity and mortality.

## Summary figure

**Table:** 

Time	Events
Pre-hospital	Patient had left lower limb claudication followed by non-healing ulcer for 3 months
Day of admission	patient presented with c/o left lower limb ulcer
Day 2	CT angiogram showed complete occlusion of the left external iliac artery with 60% stenosis in the right external iliac artery
Day 3–6	Patient was planned for left iliac artery angioplasty and underwent the procedure and developed perforation, which was managed with a covered stent
Day 7–8	Patient was discharged in a hemodynamic stable condition with antiplatelets and anticoagulants
Post discharge	Follow-up after 3 months, the patient is doing well with no symptoms, and CT shows patent stent with a good distal flow

## Case presentation

A 58-year-old male with a history of poorly controlled T2DM presented to the outpatient department of our hospital with complaints of a non-healing ulcer on the left lower limb and claudication. Physical examination revealed an afebrile status; pulse, 87 bpm, regular; blood pressure in the right upper limb in the supine position, 126/70 mmHg; respiratory rate, 16/min; saturation at room air, 99%. Cardiovascular examination revealed normal heart sounds with no murmurs. The ankle-brachial index of the left and right lower limbs was 0.4 and 1.2, respectively. Routine blood investigations revealed normal haemoglobin levels (14 g/dL; normal range: 13–17 g/dL), total leukocyte count (12,000/μL; normal range: 4500–11 000/μL), and platelet count (200 000/μL; normal range: 150 000–450 000/μL). However, renal function tests indicated mild dysfunction, with urea at 20 mg/dL (normal range: 17–43 mg/dL) and creatinine elevated at 1.7 mg/dL (normal range: 0.7–1.3 mg/dL). Blood glucose levels were markedly elevated with fasting blood sugar at 260 mg/dL (normal range: 70–100 mg/dL), postprandial blood sugar at 350 mg/dL (normal range: 70–140 mg/dL), and HbA1C at 14.2% (normal range: < 5.7%), indicating poor glycaemic control.

**Figure 1:**
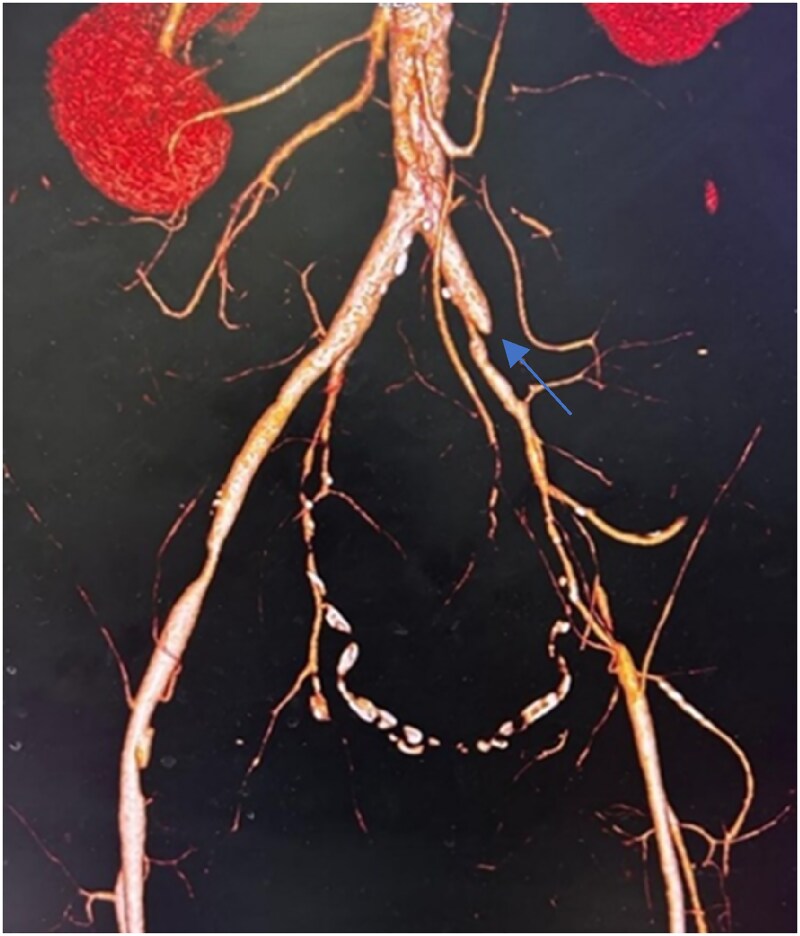
Computed tomography angiography showing complete occlusion of the left external iliac artery (arrow) with 60% stenosis in the right external iliac artery.

Computed tomography (CT) angiography revealed complete occlusion of the left external iliac artery (EIA) and 60% stenosis in the right EIA (*Figure 1*). The proximal and distal reference vessel diameters of the left EIA were 7.9 and 7.7 mm, respectively. These imaging findings guided procedural planning, including the selection of balloon and stent sizes.

Peripheral angioplasty was planned after obtaining written consent from the patient and attendant. A right femoral access was obtained using a 6F femoral sheath, and the lesion in the left EIA was crossed with a 0.035 Terumo guidewire. Predilation was performed with a 6 × 100 mm peripheral balloon at 8 atm. However, a repeat angiogram revealed haziness in the proximal EIA, which may suggest dissection, calcium, or thrombus. To address this, an 8 × 100 mm self-expanding stent (Boston Scientific, MA, USA) was deployed in the left EIA, and under-expansion was noted in the proximal stent segment. Consequently, post-dilation was performed using an 8 × 40 mm balloon at 6 atm.

**Figure 2:**
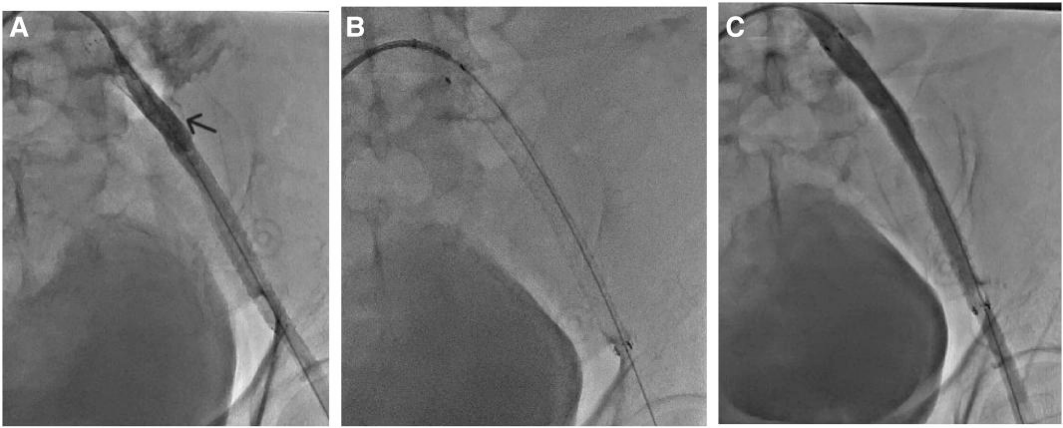
Sequence of managing iliac artery perforation. (*A*) Identification of perforation in the external iliac artery (arrow), (*B*) deployment of a covered stent, and (*C*) confirmation of successful perforation sealing with no contrast extravasation observed using cine imaging.

Immediately following this, the patient complained of back and flank pain, became haemodynamically unstable, developed hypotension, and required fluid resuscitation and norepinephrine for haemodynamic support. A repeat angiogram identified a perforation in the left proximal EIA with significant extravasation of contrast (*Figure 2A*; Supplementary material online, *Video S1*). Urgent balloon tamponade was performed using an 8 × 60 mm balloon at 2–4 atm for 15 min. Given the severity of the perforation and its location in the major vessel, a covered stent of 8 × 100 mm FLUENCY Vascular (Bard Inc., NJ, USA) was deployed to seal the perforation (*Figure 2B*), followed by post-dilation with an 8 × 40 mm balloon at 6 atm. Subsequent angiography confirmed successful perforation sealing (*Figure 2C*; Supplementary material online, *Video S2*). The patient was gradually weaned off haemodynamic support and stabilized.

**Figure 3:**
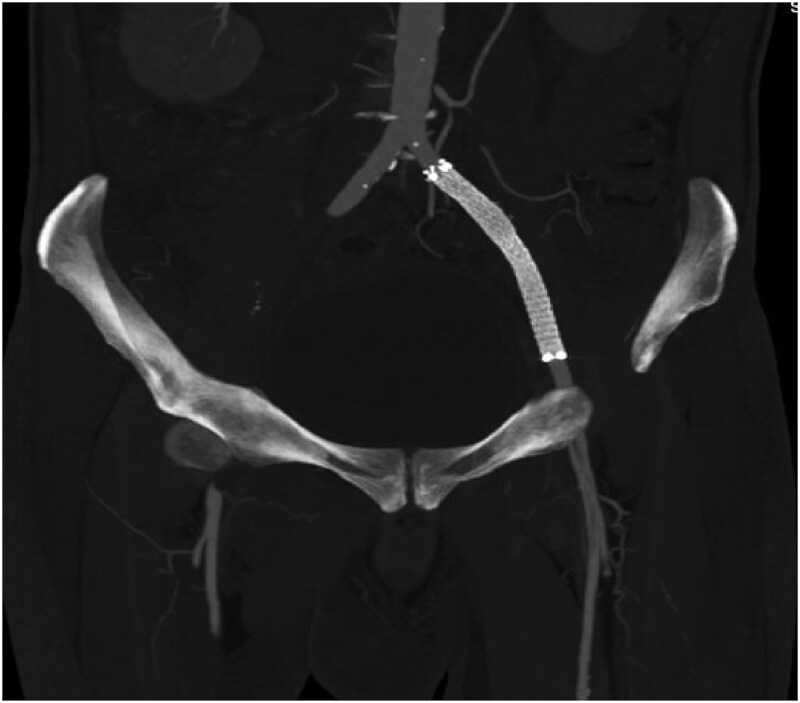
Follow-up Computed tomography angiography at three months shows the patent-covered stent in the left external iliac artery with good distal runoff.

Upon discharge, the patient was prescribed aspirin 75 mg, rosuvastatin 40 mg, and rivaroxaban 2.5 mg twice daily, in addition to oral hypoglycaemic agents and insulin, as per the endocrinologist’s advice. At the 1-month follow-up, repeat CT angiography demonstrated a patent-covered stent with good distal flow (*Figure 3*), and the patient was doing well.

## Discussion

The femoral arterial route, frequently used in peripheral angioplasty, carries risks such as local haemorrhage, groin haematoma, arteriovenous fistula, pseudoaneurysm, vessel perforation, and thrombosis. Vessel perforation often manifests as sudden hypotension, tachycardia, and shock. In particular, EIA perforation is particularly challenging due to the risk of haemorrhagic shock and death if not promptly managed.^6–10^ While rare,^4^ such complications necessitate swift and effective management to prevent adverse outcomes.

Advanced endovascular therapies have increased iatrogenic vascular access complications, including iliac artery perforation. Additionally, recent studies have highlighted an increase in vascular complications associated with alternative approaches, such as the radial route and various surgical procedures. Typical management strategies include percutaneous techniques such as balloon tamponade, stent grafts, and embolization, especially in patients with calcified atherosclerotic disease and tortuous vessels.^4,11^ Endovascular therapy for traumatic and iatrogenic vascular complications, including iliac artery ruptures and injuries, has shown success with approaches like stent grafts and covered stents, improving management as endoluminal techniques evolve.

In this case, CT angiography was used to measure vessel diameters, which guided device selection and balloon sizing. Operators should meticulously plan interventions based on preprocedural imaging to reduce complications and improve procedural success. Although intravascular imaging and advanced techniques like scoring balloons and atherectomy devices were not utilized here, they remain critical tools for optimizing vascular bed preparation and minimizing risk in calcified lesions. Future cases could benefit from incorporating these techniques.

In conclusion, this case highlights the critical role of early detection and timely intervention in managing severe complications during peripheral angioplasty. The successful use of a covered stent in this instance not only prevented further complications but also exemplified the effectiveness of standardized, guideline-based approaches in ensuring patient safety. This case adds to the growing body of evidence supporting the use of covered stents in vascular interventions and underscores the need for continuous vigilance and preparedness in managing high-risk patients undergoing complex endovascular procedures.

## Supplementary Material

ytaf126_Supplementary_Data

## Data Availability

The data underlying this article will be shared on reasonable request to the corresponding author.

## Lead author biography

Dr Saroj Kumar Sahoo completed his MBBS at MKCG Medical College in Berhampur. He pursued his MD in Internal Medicine and DM in Cardiology at PGIMER, Chandigarh. Presently, he serves as an Assistant Professor at AIIMS in Bhubaneswar, Odisha. His area of interest encompasses interventional cardiology, particularly complex coronary interventions such as bifurcation stenting, chronic total occlusion (CTO), acute coronary syndrome (ACS), and primary percutaneous coronary intervention (PCI).

## Notes

**Consent:** The authors confirm that written consent for submission and publication of this case report, including images and associated text, has been obtained from the patient in line with COPE guidance.

**Funding:** No funding was received for the preparation of this case report.
